# DCNN for Pig Vocalization and Non-Vocalization Classification: Evaluate Model Robustness with New Data

**DOI:** 10.3390/ani14142029

**Published:** 2024-07-09

**Authors:** Vandet Pann, Kyeong-seok Kwon, Byeonghyeon Kim, Dong-Hwa Jang, Jong-Bok Kim

**Affiliations:** Animal Environment Division, National Institute of Animal Science, Rural Development Administration, Wanju 55365, Republic of Korea; pannvandet@korea.kr (V.P.); kskwon0512@korea.kr (K.-s.K.); osorikim619@korea.kr (B.K.); dh5210@korea.kr (D.-H.J.)

**Keywords:** audio classification, audio feature extraction, pig vocalization, smart farming, audio data augmentation, machine learning, deep learning model, convolutional neural networks (CNNs), smart livestock farming, environmental animal

## Abstract

**Simple Summary:**

This study addresses the significance of animal sounds as valuable indicators of both behavior and health in animals, emphasizing the challenges involved in collecting datasets for deep learning models. Particularly, in the context of classifying pig vocalization and non-vocalization, it is identified as laborious and time-consuming when relying on human efforts. In response to these challenges, the research proposes a new approach utilizing a deep learning model to automatically classify pig vocalization and non-vocalization with high accuracy. The success of this method not only provides an efficient means of collecting pig sound datasets but also presents a promising avenue for improving the classification of pig vocalization and non-vocalization in deep learning models, thereby contributing to advancements in animal behavior research and health monitoring.

**Abstract:**

Since pig vocalization is an important indicator of monitoring pig conditions, pig vocalization detection and recognition using deep learning play a crucial role in the management and welfare of modern pig livestock farming. However, collecting pig sound data for deep learning model training takes time and effort. Acknowledging the challenges of collecting pig sound data for model training, this study introduces a deep convolutional neural network (DCNN) architecture for pig vocalization and non-vocalization classification with a real pig farm dataset. Various audio feature extraction methods were evaluated individually to compare the performance differences, including Mel-frequency cepstral coefficients (MFCC), Mel-spectrogram, Chroma, and Tonnetz. This study proposes a novel feature extraction method called Mixed-MMCT to improve the classification accuracy by integrating MFCC, Mel-spectrogram, Chroma, and Tonnetz features. These feature extraction methods were applied to extract relevant features from the pig sound dataset for input into a deep learning network. For the experiment, three datasets were collected from three actual pig farms: Nias, Gimje, and Jeongeup. Each dataset consists of 4000 WAV files (2000 pig vocalization and 2000 pig non-vocalization) with a duration of three seconds. Various audio data augmentation techniques are utilized in the training set to improve the model performance and generalization, including pitch-shifting, time-shifting, time-stretching, and background-noising. In this study, the performance of the predictive deep learning model was assessed using the k-fold cross-validation (k = 5) technique on each dataset. By conducting rigorous experiments, Mixed-MMCT showed superior accuracy on Nias, Gimje, and Jeongeup, with rates of 99.50%, 99.56%, and 99.67%, respectively. Robustness experiments were performed to prove the effectiveness of the model by using two farm datasets as a training set and a farm as a testing set. The average performance of the Mixed-MMCT in terms of accuracy, precision, recall, and F1-score reached rates of 95.67%, 96.25%, 95.68%, and 95.96%, respectively. All results demonstrate that the proposed Mixed-MMCT feature extraction method outperforms other methods regarding pig vocalization and non-vocalization classification in real pig livestock farming.

## 1. Introduction

Pork holds significant economic importance and has been a vital source of human nutrition [1]. It has remained the most widely consumed meat globally for an extended period, and hundreds of millions of individuals from all corners of the world continue to favor it [2]. China occupies a notable position as a leading producer and consumer of pork worldwide [3]. In 2021, pork production in China reached approximately 52.96 million tons, representing over 58.9% of total meat production [4]. According to the USDA (United States Department of Agriculture) reports, China is expected to remain the largest pork consumer, holding nearly 46% globally. The European Union and United States will have 14% and 8.4% shares, respectively. Southeast Asia anticipates the highest growth in pork consumption at 20.8% of the world share by 2031 [5]. The enormous pork demand has driven the rapid expansion of industrial-scale pig farming operations, which has created a need for precision livestock farming (PLF) technologies instead of traditional methods to meet increasingly stringent standards [6,7].

PLF enables the expansion of the livestock sector towards sustainable production by integrating production and animal health considerations, facilitating optimal animal stocking densities and prompt disease management, and establishing more efficient production models [8]. Because large-scale breeding facilities have high feeding densities, the health status of pigs becomes a high-priority problem for farmers [9]. Therefore, farmers require assistance in properly caring for each pig and quickly identifying anomalies using modern technologies [10]. Currently, PLF plays a crucial role in managing the welfare of modern group-pig livestock [11]. Many researchers have investigated the efficiency of the PLF to address how PLF enhances the welfare management of animal farms [12,13,14]. There are PLF systems used in pig farms to monitor pig health and welfare, such as pig identification, pig automated weight detection, pig behavior, and vocalization monitoring [15]. Pig vocalization is essential for delivering real-time health information, improving the assessment of sick pigs, controlling the environment, and encouraging effective and healthy breeding methods.

More than twenty years ago, research on pig vocalization was conducted to investigate the welfare of piglets [16,17]. Many scholars regard pig vocalization as an effective tool for assessing the well-being and health of pigs [18,19,20]. Hillmann et al. [21] used pig vocalization to analyze pig behavioral adaptation to ambient temperatures. The authors used a video recorder and an external microphone to collect data only during nighttime to avoid management activities during the daytime. Guarino et al. [22] proposed an algorithm for detecting pig coughing and defining the condition of the health of the pig. This study used the manpower to collect the coughing sound by standing next to the pigs. Many researchers have applied deep learning models based on DCNN to address the health condition using pig sound datasets. Research about pig coughing recognition based on DCNN was introduced by Yanling Yin et al. [23]. The audio dataset was collected from the pig pen and converted to spectrogram images. The authors proposed an algorithm using spectrogram images as the input into a pre-trained deep learning model. Weizheng Shen et al. proposed two different feature fusion methods in their two published papers using deep feature information extracted from the CNN network to recognize the pig coughing [24,25]. Wang et al. [26] introduced a lightweight CNN model for recognizing the estrous sound of the sows. They collected an audio dataset by holding a digital recorder to record sow sounds in the pig barn. The challenges in collecting pig sound data using recording devices include distinguishing vocalization from non-vocalization sound within the recorded audio files, requiring significant time and manpower. On the other hand, some farms restrict human access due to animal safety concerns. Obtaining permission to collect sound data and navigate these restrictions may pose a barrier to researchers or data collectors.

The observations of this study indicate that deep learning methods have played an important role in the study of pig vocalizations. However, enormous amounts of data are required to build a high-efficiency deep learning model, and gathering these data is still traditional and time-consuming.

The main contributions are summarized as follows:

- Design a new pig vocalization and non-vocalization classification model using deep learning network architecture and audio feature extraction methods.
- Implement various audio feature extraction methods and compare the classification performance results using a deep learning model.
- Propose a novel feature extraction method to enrich the input information that can improve the classification accuracy of the model. This proposed method is robust enough to classify pig vocalization and non-vocalization in different data collection environments.
- Create datasets of pig vocalization and non-vocalization to handle the problem of insufficient data.
- Compare the performance of the various audio feature extraction methods. The proposed method improves the classification performance and efficiently classifies pig vocalization and non-vocalization.

## 2. Materials and Methods

### 2.1. Data Acquisition

**Figure 1:**
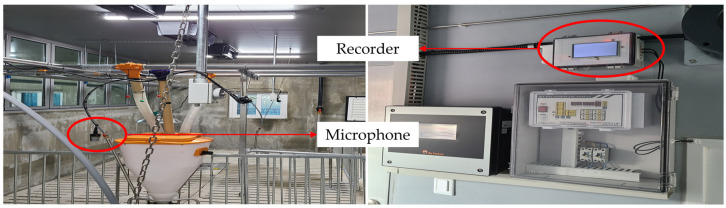
The installation of recording devices inside the pig farm.

Three collections of pig audio datasets named Nias, Gimje, and Jeongeup were systematically collected from three domestic pig farms in Jeonju City, Gimje City, and Jeongeup City, respectively. These three cities are located in Jeonbuk-do Province, Republic of Korea. A high-quality PLM-Q5 noise reduction microphone with a frequency range of 20 Hz to 20 kHz was strategically positioned at a height of 150 cm above the ground, with an average of 12 pigs per pen. The recording sample rate was 44,100 Hz, with a resolution of 16 bits. The recording apparatus utilized for this study was the Raspberry Pi 4 Model B Rev 1.5, enabling continuous recording for 24 h. Each recording file was saved at hourly intervals. These comprehensive datasets provide detailed auditory profiles of the pig farms, capturing ambient sounds and pig vocalization. Figure 1 shows the installation of the devices for pig audio data collection in a pig farm.

### 2.2. Data Preprocessing

As mentioned in Section 2.1, during the dataset collection period, continuous recordings were conducted for a duration of 24 h, with each recording file saved at one-hour intervals. To enhance the precision of the dataset for model training, the audio recordings were further processed by trimming them into 3 s intervals. In a pig farm, the acoustic environment is complex, featuring a wide range of sounds, including various pig vocalization and background noises. To categorize these sounds, the trimmed pig audios were classified by manually annotating them, a process carried out in collaboration with animal science researchers. The audio files were classified into two sets based on the presence of pig sounds: the pig vocalization set included all files containing pig sounds, while the pig non-vocalization set included those without pig sounds. Audio files with noises from human activities such as human voices and cleaning activities were discarded. Human evaluators conducted the selection process to ensure a representative and diverse set of audio samples, providing a comprehensive dataset encompassing various aspects of the pig farm environment. Following this step, a curated selection of 4000 WAV audio files in each farm was meticulously chosen and labeled, with a balanced distribution of 2000 files containing pig vocalizations and 2000 files containing non-vocalization. The datasets contain different background noise and sound loudness. The amplitude levels of the dataset are measured using decibels relative to the full-scale (dBFS) unit. All collections of pig audio datasets and dBFS measurements are listed in Table 1.

### 2.3. Audio Data Augmentation

Acquiring a large and diverse pig dataset to train a deep learning model is challenging. In this study, data augmentation is utilized to increase the size of the training set and the diversity of the dataset artificially [27]. This technique improves deep learning models and makes them more robust and generalizable to variations in real-world data. This study experiments with four audio data augmentation techniques: pitch-shifting, time-shifting, time-stretching, and background noise. Each technique generates 3200 new audio samples to the original training set. In total, the training set contains 16,000 samples for model training.

- Pitch-shifting is a digital signal processing technique that alters the pitch of an audio signal without changing its duration [28]. Each audio sample was pitch-shifted by random values from 0 to 4 and 12 bins per octave.
- Time-shifting involves displacing audio to the left or right by a randomly determined duration. When shifting audio to the left (forward) by *x* seconds, the initial *x* seconds were designated 0. Conversely, when shifting audio to the right (backward) by *x* seconds, the last *x* seconds were designated 0.
- Time-stretching involves adjusting the speed of the audio sample, either slowing it down or speeding it up without affecting the pitch of the sound. In this study, each sample underwent time stretching using a stretch factor of 1.0.
- Background-noising is an intentional addition of background noise to an audio sample. In this study, each audio sample was added with white noise. Each background-noising *z* was calculated using *z* = *x* + *w*·*y*, where *x* represents the audio signal of the original sample, *y* denotes the signal with the background scene, and *w* serves as a weighting parameter. Notably, the weighting parameter *w* has been selected from a uniform distribution randomly in the range of [0.0, 1.0].

**Figure 2:**
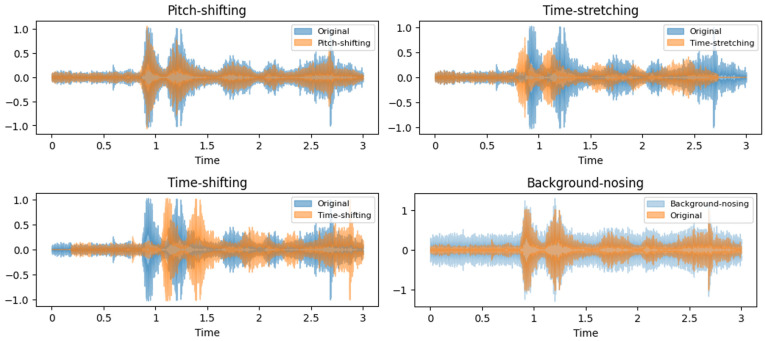
Data augmentation visualization in the wave signals of each method.

Samples of the audio augmentation datasets are visualized in Figure 2.

### 2.4. Audio Feature Extraction

Raw audio signals are complex and high-dimensional, making it difficult for deep learning networks to process them directly. Audio feature extraction is essential when working with deep learning network models. It condenses raw audio data into a lower-dimensional representation that captures important characteristics from an audio signal, allowing the network to learn more efficiently. Recently, audio feature extraction methods have been widely used to extract useful features from audio signals. Rezapour Mashhadi et al. [29] used these various types of audio feature extraction methods in their research on speech emotion recognition. The authors mentioned that these methods are useful for extracting various speech signal characteristics for their model. Gupta Saurabh et al. [30] studied animal speech emotion recognition using a deep learning model. They employed the MFCC and Chroma methods to extract the major features from animal sounds. The authors stressed that these feature extraction methods provide useful feature relationships from audio signals that benefit model classification. Another current study about depression detection also utilized MFCC to extract speech audio for their model [31]. By observing the significance of these methods, this study introduces four feature extraction methods: MFCC, Mel-spectrogram, Chroma, and Tonnetz. In addition, a novel audio feature extraction method called Mixed-MMCT is proposed in this research work to enhance the performance of the classification accuracy of the model. The details of each method are described in the next section. 

#### 2.4.1. MFCC (Mel-Frequency Cepstral Coefficients)

MFCC is a widely used audio feature extraction technique in speech and audio classification [30,32,33]. It captures the essential characteristics of the audio signal, making it particularly useful for audio classification. The processing of MFCC includes segmenting the audio signal into short frames and applying the window function to each frame. It is followed by performing the fast Fourier transform (FFT) on each audio signal, taking the logarithm of the magnitude of the FFT. The resulting energy is then passed through a Mel filter to obtain Mel filter energy. As a result, 20-dimensional MFCC coefficients are obtained after calculating the discrete cosine transform (DCT) to the log Mel filter energy.

#### 2.4.2. Mel-Spectrogram

The Mel-spectrogram represents the short-term power spectrum of an audio signal as it evolves over time, converted to the Mel-frequency scale [34]. Converting a raw audio signal into a Mel-spectrogram involves several crucial steps. First, a pre-emphasis filter is applied to enhance high-frequency components, achieved by subtracting a fraction of the previous sample from the current one. The pre-emphasized signal is then divided into short, overlapping frames through farming, followed by applying a Hamming window function to mitigate spectral leakage. Within a sample window input of three seconds long, the sampling rate is 22,050 Hz, the fast Fourier transform (FFT) window size is 2048, and the hop length is 512 each time. The FFT calculates the discrete Fourier transform (DFT) for each window frame, transitioning the signal from the time domain to the frequency domain. Subsequently, the Mel-filterbank is applied to the power spectrum obtained from the FFT, transforming the signal into the equal Mel-frequency scale with 128 Mel bands. Finally, a logarithmic transformation, emulating human auditory perception, is performed by taking the logarithm of the filterbank energies.

#### 2.4.3. Chroma

Chroma audio feature extraction focuses on capturing the pitch content or tonal information of an audio signal [35]. Many researchers utilize chroma features as the input for deep learning models to address audio detection and recognition problems [36,37,38]. The chroma feature represents the distribution of pitch classes that are typically manifested as a 12-element feature vector that illustrates the presence of energy for each pitch class in the signal [39]. The Short-Term Fourier Transform (STFT) is applied to convert the raw audio signal into the frequency domain, slicing it into short, overlapping frames. Within each slice, chroma calculates the strength of each pitch class, essentially summing the energy within the specific frequency ranges corresponding to each pitch class. Following this, to account for differences in overall loudness, the chroma is then normalized. Finally, a logarithmic transformation is applied to the chroma values, mimicking the human perception of pitch, which tends to follow a more logarithmic pattern than a linear one.

#### 2.4.4. Tonnetz

Tonnetz audio feature extraction focuses on capturing tonal relationships and harmonic content in audio signals. Many researchers use the Tonnetz feature to address acoustic and music detection [40,41,42]. Like chroma features, the raw audio signal is transformed into the frequency domain using STFT, which divides the signal into short, overlapping frames. Since Tonnetz first requires chroma features, in each frame of the STFT, chroma calculates the energy within specific frequency bands corresponding to each pitch class. Subsequently, the Tonnetz features are computed based on the chroma features by capturing the tonal centroid and spreading pitch classes.

#### 2.4.5. Mixed-MMCT

**Figure 3:**
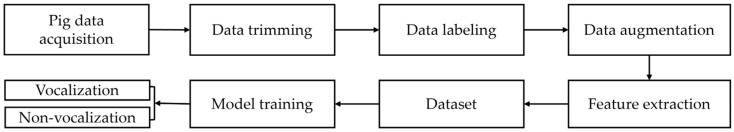
The overall flow diagram of the classification method.

**Figure 4:**
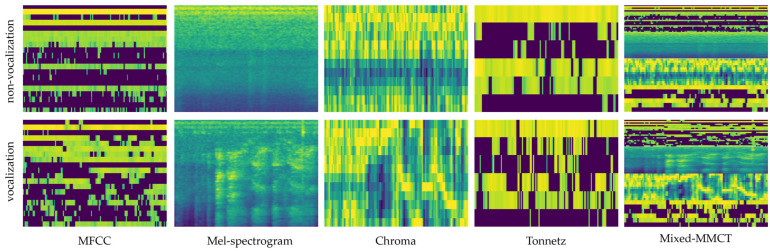
The visualization of the pig vocalization sample in each feature extraction method. The first row displays the non-vocalization samples, and the second row shows the vocalization samples.

Mixed-MMCT is a novel audio feature extraction method introduced in this study. It is a new feature name formed by mixing the first letters of Mel-spectrogram, MFCC, Chroma, and Tonnetz. The objective of this method is to improve the pig vocal classification accuracy by concatenating Mel-spectrogram, MFCC, Chroma, and Tonnetz features along the appropriate axis to create a single, combined feature vector for each time frame. Combining these features creates a comprehensive and rich representation of audio data, capturing different aspects of the sound signal. This new feature extraction method outperforms individual methods, particularly in the context of pig vocalization classification. Figure 3 shows the overall flow diagram of the classification method, and Figure 4 visualizes the spectrogram form of the pig vocalization sample in each audio feature extraction method.

### 2.5. Deep CNN Architecture

**Figure 5:**
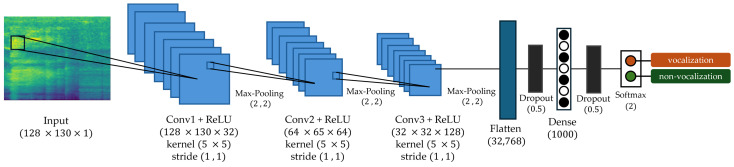
The overall structure of the proposed deep learning network architecture model.

Figure 4 shows the overall proposed network architecture diagram. The input size of the network follows the output size of the audio feature extraction in each method. The input sizes of MFCC, Mel-spectrogram, Chroma, Tonnetz, and Mixed-MMCT are 20×130×1, 128×130×1, 12×130×1, 12×130×1, and 166×130×1, respectively. The network backbone architecture consists of three convolutional layer blocks. The sizes of the convolutional layer filter in the first, second, and third blocks are 32, 64, and 128, with a stride of 1×1 and a kernel size of 5×5, respectively. Different filter and kernel sizes were experimented with, and the filter and kernel sizes used in the experiments outperformed others in terms of performance and accuracy. A 2×2 max-pooling layer is applied to down sampling, and the Rectified Linear Units (ReLUs) are used to implement nonlinear activation functions. The ReLUs function $f\left(x\right)$ is calculated using $f\left(x\right)=\max(0,x)$, where x represents the input and max⁡(0,x) retains only values greater than 0. Dropout [43] is applied after the last layer to prevent over-fitting. Two fully connected layers are applied. The first fully connected layer has 1000 neurons, and the second fully connected layer is connected to a softmax function with two neurons and outputs the probability for each class. It is classified as vocalization when the output value of the first neuron is greater than the output value of the second neuron; otherwise, it is non-vocalization. Figure 5 shows the overall proposed network architecture diagram.

### 2.6. Experimental Setting

Initially, this work follows [44] to resample the monophonic signal to the default sample rate of 22,050 Hz in the data preprocessing. The dropout parameter is set to 0.5, and the batch size is 16. During the training process, the initial learning rate is set to 5 × 10^−4^, the decay step is 1000, and the decay rate is 0.9. In addition, the stochastic gradient descent (SGD) optimizer is applied as the model optimizer with a momentum of 0.9. The Rectified Linear Units (ReLUs) are used to implement nonlinear activation functions. The overall model is trained up to 50 epochs to complete each experiment. All experiments in this study are implemented using Python programming language and a TensorFlow-based open-source [45] deep learning framework. The model is trained on a Windows 10 operating system with an NVIDIA GeForce RTX 2080 Ti GPU to benefit from faster training times in deep learning frameworks with the support of Cuda and cuDNN. The CPU is an Intel(R) Core (TM) i7-8700 CPU with six cores operating at 3.30 GHz.

### 2.7. Evaluation Criteria

In this study, the performance of a predictive model is assessed using the four evaluation parameters: accuracy, precision, recall, and F1-score.

- Accuracy serves as an intuitive performance metric specifically designed to characterize the effectiveness of an algorithm in classification tasks. It qualifies the ratio of correctly predicted samples to the overall sample count, as demonstrated by Equation (1).
  Accuracy = (TP + TN)/(TP + TN + FP + FN)(1)
- Precision is a metric focused on evaluating the accuracy of positive predictions. Specifically, it calculates the precision for the minority class, representing the ratio of correctly predicted positive samples to the total predictive positive samples. The computation of precision is outlined in Equation (2).
  Precision = TP/(TP + FP)(2)
- Recall is a metric that calculates the number of accurate positive predictions made among all possible positive predictions. In contrast to precision, which focuses solely on accurate positive predictions out of all positive predictions, recall encompasses a broader scope. The computation of recall is defined in Equation (3).
  Recall = TP/(TP + FN)(3)
- F1-score provides a consolidated measure by combining precision and recall into a single metric encompassing both aspects. It has the ability to convey scenarios with high precision and poor recall, as well as situations with poor precision and perfect recall. The computation of the F1-score is outlined in Equation (4).
  F1-score = 2 × (Precision × Recall)/(Precision + Recall)(4)
  where true positive (TP) signifies a correctly classified positive sample, true negative (TN) denotes the number of predictions accurately identifying the sample as negative, false positive (FP) represents the number of samples wrongly classified as positive, and false negative (FN) refers to the quantity of samples inaccurately identified as negative.

## 3. Results

### 3.1. Experimental Results

**Figure 6:**
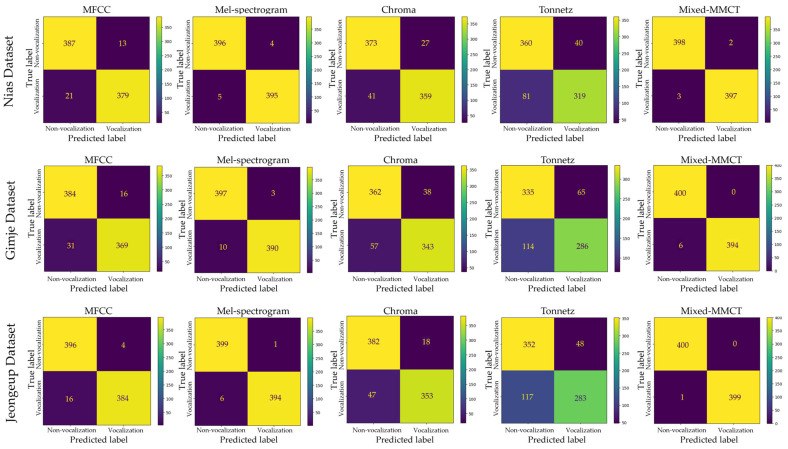
Confusion matrix of the classification results of the different feature extraction methods.

**Figure 7:**
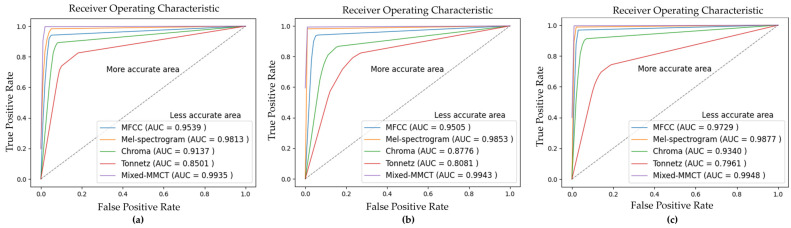
The ROC curve visualization of the model classification performance in each feature extraction method. (**a**–**c**) are ROC curves of Nias, Gimje, and Jeongeup, respectively.

This section reports all of the model evaluation results. This study performed experiments in the classification task and used the fivefold cross-validation technique to assess the predictive model by dividing the dataset into five partitions: four partitions are reserved for the training set, whereas the remaining partition is reserved as a validation set. The model repeated training five times and used the average of the five validation results to measure classification accuracy. All the evaluation results are displayed as the percentage of the right predictions. The confusion matrixes of the pig vocalization and non-vocalization classification results in each method are illustrated in Figure 6. A confusion matrix displays the performance of the classifier in the experiment. According to the confusion matrices in Figure 6, the model can correctly classify the true positive (TP) and true negative (TN) with the proposed Mixed-MMCT method compared to other methods. However, the classification rate drops considerably with the Tonnetz method. Table 2 shows the measurements of the performance of the model with accuracy, precision, recall, and F1-score metrics in each dataset. The average accuracy of fivefold cross-validation on three databases, namely, Nias, Gimje, and Jeongeup, across various feature extraction methods, including MFCC, Mel-spectrogram, Chroma, Tonnetz, and Mixed-MMCT, yielded consistent results. Specifically, for the Nias dataset, the accuracies were 95.44%, 98.25%, 91.41%, 85.03%, and 99.50%, respectively. Similarly, for the Gimje dataset, the accuracies were recorded as 95.06%, 98.78%, 87.72%, 80.78%, and 99.56% across the same feature extraction methods. Lastly, for the Jeongeup dataset, the accuracies for the same feature extraction methods were 97.34%, 98.87%, 93.44%, 79.66%, and 99.67%, respectively. The results demonstrate that the proposed Mixed-MMCT feature extraction method achieved the highest performance compared to other methods regarding pig vocalization and non-vocalization classification tasks. The Receiver Operating Characteristic (ROC) curve is employed to evaluate the model classification of each method. The ROC curves are illustrated in Figure 7. 

As reported in Figure 7, the Mixed-MMCT method exhibits the highest area under the curve. This indicates that, in this scenario, the classifier demonstrates superior performance in distinguishing both positive and negative samples.

### 3.2. Robustness Experimental Results

**Figure 8:**
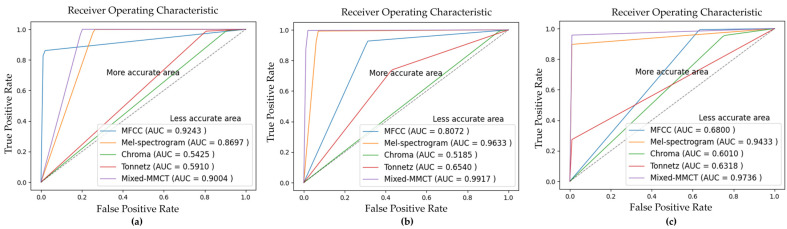
The ROC curve visualization of the model robustness classification performance. (**a**–**c**) are the ROC curves of NGdb, NJdb, and GJdb, respectively.

**Figure 9:**
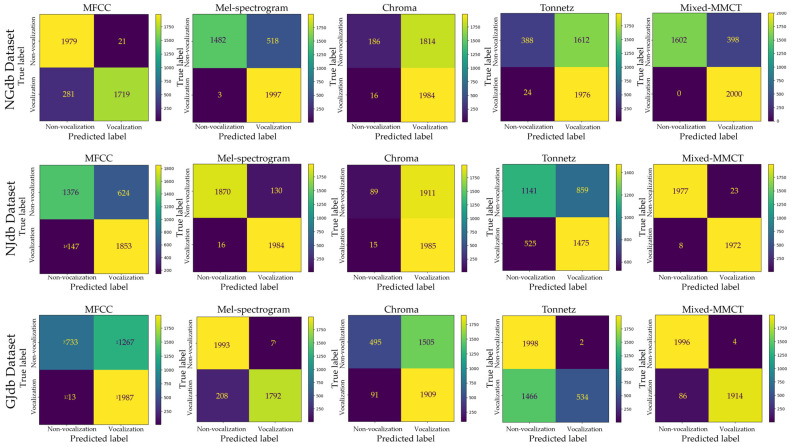
Confusion matrix results of the model robustness classification performance.

Robustness experiments were performed to prove the effectiveness of the model. As mentioned in Section 2.1, three datasets were gathered from three different actual pig farms. Hence, to conduct model robustness experiments, three datasets, namely, NGdb, NJdb, and GJdb, were created by combining pairs of datasets: Nias with Gimje, Nias with Jeongeup, and Gimje with Jeongeup, respectively. Each combined dataset comprised 8000 samples. These datasets were utilized for training and testing purposes, with one dataset used for training and the remaining dataset used for testing in each combination. Specifically, NGdb was employed for training, with Jeongeup as the testing set; NJdb served as the training set, while Gimje was used for testing; GJdb was utilized for training, with Nias designated as the testing set. All data preprocessing and experimental settings, including the data augmentation, input size, batch size, learning rate, and training epoch, were applied to match the previous experiments. Table 3 shows the measurements of the performance of the model with accuracy, precision, recall, and F1-score metrics in each experiment. Table 4 summarizes the average performance results of the robustness experiments. It can be seen that the model works better with the Mixed-MMCT method than with other methods. Figure 8 and Figure 9 show ROC curves and confusion matrices, respectively.

## 4. Discussion

This study aimed to classify pig vocalization and non-vocalization and to help with the pig sound data collection task. To achieve this goal, this study constructed a new deep learning network architecture and examined various audio feature extraction methods. In the process of audio data collection, this study only labeled those audio sounds that contained pig sounds and no pig sounds as vocalization and non-vocalization, respectively—in other words, those pig sounds that were visible or invisible on the waveform and distinguishable by the human ear. This can make the classification performance slightly superior to real-time classification performance.

The different feature extraction methods, including MFCC, Mel-spectrogram, Chroma, and Tonnetz, captured various aspects of the audio signal. Comparing these methods allowed this study to assess their effectiveness in representing the relevant information. Furthermore, a new feature extraction method, Mixed-MMCT, was introduced to improve the model performance accuracy. When discussing the output size of the feature extraction method, this study carefully selected that which was inspired by previous research [46,47,48,49]. As visualized in Figure 4, each feature produced different characteristics of an audio signal over time. Mel-spectrogram provided feature information about the distribution of energy across different frequency bands with 128 mel-scales. MFCC was derived from the Mel-spectrogram, obtaining 20 cepstral coefficients representing the spectral characteristics of the audio signal. The Chroma method mapped the magnitude spectrum of the audio signal onto the 12-dimensional vectors, while Tonnetz computed the tonal centroid features and produced only six-dimensional basis features. As shown in Table 2, the different types of feature extraction methods have a clear influence on the model performance. These reports show that the methods that produce smaller sizes of the audio features perform poorer than the methods that produce bigger sizes of the audio features. For instance, the model does not work well with Chroma and Tonnetz. In contrast, the model achieves excellent performance with MFCC and Mel-spectrogram. Additionally, the combination of the feature extraction method called Mixed-MMCT yields superior performance and demonstrates other evidence, as reported in [1,48,50,51,52]. Obviously, feature extraction methods that generate large audio features may have drawbacks related to computational complexity. Table 5 summarizes the floating-point operations per second (FLOPS), the number of trainable parameters, and the inference time for each method in a single input image for comparison.

To further prove the effectiveness of the model, this model is supposed to compare the performance with the existing models or conduct more experiments with the existing dataset. However, at the time of this study, there is no publicly available pig vocalization and non-vocalization benchmark dataset. Therefore, model robustness experiments were conducted to verify the model performance. As described in Section 3.2, two datasets were combined to create a training set, and the remaining dataset was used as a test set for model robustness experiments. Table 3 shows that the MFCC features extraction method outperforms other methods when the model trains with NGdb. Based on this scenario, the MFCC method can extract rich information with data that have a high dBFS, or the higher dBFS of the test set might have a positive effect on the performance. However, the MFCC performs poorly when the model tests with other test sets, while the Mixed-MMCT maintains the performance at a high score. As shown in Table 4, the Mixed-MMCT method continued to demonstrate outstanding performance on a new dataset in mode robustness experiments. The Mel-spectrogram method showed slightly decreased performance, while the Chroma and Tonnetz methods decreased remarkably, making the model nearly impossible to classify with the new dataset. From the observations in this study, two main reasons caused the model to drop its performance. The first main reason is the dataset variation of each farm. The acoustic characteristics of the audio signal vary with different conditions and environments, such as background noise, reverberation, and the age of pigs. The second main reason is audio input features. The more information the feature extraction method obtains, the better the deep learning model performs, and vice versa.

Finally, this study indicates which audio feature extraction method is suitable for generating the input for training deep learning models to classify pig vocalization and non-vocalization in actual pig farms. The results demonstrate that combining feature methods improves the model performance compared to using them separately. The findings in this study will be used in data collection to separate pig vocalization and non-vocalization automatically for future work.

## 5. Conclusions

This study implements audio feature extraction methods with a deep learning network to solve pig vocalization and non-vocalization problems. Data augmentation techniques are employed for model training to tackle the issue of an insufficient training dataset. These techniques help to improve the performance and generalization of the model. Furthermore, this study introduces a new audio feature extraction method to enhance model classification accuracy by combining many other feature extraction methods. Consequently, this new method provides superior performance compared to other methods.

The results of this study may become a significant and useful solution to the pig vocalization data collection problem. In future work, pig vocalization classifications such as screaming, grunting, squealing, and coughing will be considered. This future research will provide solutions for improving animal welfare monitoring in pig farms. 

## Figures and Tables

**Table 1:** Summary of the pig audio dataset used for model training and evaluation.

Dataset	Type	Growth Stage	Amount	Min dBFS	Max dBFS	Average dBFS
Nias	Vocalization	Growing fattening (30–110 kg)	2000	−36.56	−6.98	−24.86
	Non-Vocalization		2000	−37.97	−24.01	−28.58
Gimje	Vocalization	Weaning (5–30 kg)	2000	−35.74	−9.03	−26.07
	Non-Vocalization		2000	−39.26	−21.08	−29.30
Jeongeup	Vocalization	Fattening (60–110 kg)	2000	−23.60	−5.96	−18.99
	Non-Vocalization		2000	−24.72	−18.98	−22.69

**Table 2:** The average results (%) of the accuracy, precision, recall, and F1-score using a fivefold cross-validation technique with the Nias, Gimje, and Jeongeup datasets.

Dataset	Methods	Accuracy	Precision	Recall	F1-Score
Nias	MFCC	95.44	95.48	95.43	95.46
	Mel-spectrogram	98.25	98.29	98.23	98.26
	Chroma	91.41	91.51	91.39	91.45
	Tonnetz	85.03	85.33	85.02	85.17
	Mixed-MMCT	**99.50**	**99.51**	**99.50**	**99.50**
Gimje	MFCC	95.06	95.08	95.07	95.07
	Mel-spectrogram	98.78	98.79	98.78	98.79
	Chroma	87.72	87.86	87.77	87.81
	Tonnetz	80.78	81.11	80.81	80.96
	Mixed-MMCT	**99.56**	**99.56**	**99.57**	**99.57**
Jeongeup	MFCC	97.34	97.35	97.35	97.35
	Mel-spectrogram	98.87	98.87	98.88	98.87
	Chroma	93.44	93.59	93.42	93.51
	Tonnetz	79.66	80.29	79.62	79.95
	Mixed-MMCT	**99.67**	**99.65**	**99.66**	**99.66**

The bold values denote the optimal value, improving the visual result comparison.

**Table 3:** The average robustness experiment results (%) of the accuracy, precision, recall, and F1-score using a fivefold cross-validation technique with the NGdb, NJdb, and GJdb datasets.

Training Set	Test Set	Methods	Accuracy	Precision	Recall	F1-Score
NGdb (Nias + Gimje)	Jeongeup	MFCC	**92.45**	**93.18**	**92.45**	**92.81**
		Mel-spectrogram	86.98	89.60	86.98	88.27
		Chroma	54.25	72.16	54.25	61.94
		Tonnetz	59.10	74.62	59.10	65.96
		Mixed-MMCT	90.05	91.70	90.05	90.87
NJdb (Nias + Jeongeup)	Gimje	MFCC	80.72	82.58	80.73	81.64
		Mel-spectrogram	96.35	96.50	96.35	96.42
		Chroma	51.85	68.26	51.85	58.93
		Tonnetz	65.40	65.84	65.40	65.62
		Mixed-MMCT	**99.22**	**99.23**	**99.23**	**99.23**
GJdb (Gimje + Jeongeup)	Nias	MFCC	68.00	79.66	68.00	73.37
		Mel-spectrogram	94.62	95.08	94.63	94.85
		Chroma	60.10	70.19	60.10	64.75
		Tonnetz	63.30	78.65	63.30	70.15
		Mixed-MMCT	**97.75**	**97.83**	**97.75**	**97.79**

The bold values denote the optimal value, improving the visual result comparison.

**Table 4:** The average results (%) of all the robustness experiments.

Methods	Accuracy	Precision	Recall	F1-Score
MFCC	80.39	85.14	80.39	82.70
Mel-spectrogram	92.65	93.73	92.65	93.19
Chroma	55.40	70.20	55.40	61.93
Tonnetz	62.60	73.04	62.60	67.42
Mixed-MMCT	**95.67**	**96.25**	**95.68**	**95.96**

The bold values denote the optimal value, improving the visual result comparison.

**Table 5:** Comparison of the method computational complexity per signal input image.

Methods	GFLOPS	Trainable Parameters (M)	Inference Time (s)
MFCC	0.145	4.36	0.031
Mel-spectrogram	0.939	33.03	0.039
Chroma	0.086	2.30	0.023
Tonnetz	0.086	2.30	0.023
Mixed-MMCT	1.21	41.22	0.046

## Data Availability

The data presented in this study are available on request from the corresponding author. The data are not publicly available due to being part of an ongoing research study and due to privacy. Code availability for access: https://github.com/codextivity/Pig-Vocalization-and-Non-Vocalization-Classification (accessed on 3 July 2024).
